# Effect of UV-C Irradiation and Lactic Acid Application on the Inactivation of *Listeria monocytogenes* and Lactic Acid Bacteria in Vacuum-Packaged Beef

**DOI:** 10.3390/foods10061217

**Published:** 2021-05-28

**Authors:** Giannina Brugnini, Soledad Rodríguez, Jesica Rodríguez, Caterina Rufo

**Affiliations:** 1 Instituto Polo Tecnológico de Pando, Facultad de Química, Universidad de la República, By Pass de Pando y Ruta 8, Pando 91000, Uruguay; gbrugnini@fq.edu.uy (G.B.); srodriguez@fq.edu.uy (S.R.); jsrodriguez@fq.edu.uy (J.R.); 2 Graduate Program in Chemistry, Facultad de Química, Universidad de la República, General Flores 2124, Montevideo 11800, Uruguay

**Keywords:** beef, lactic acid, UV-C, *Listeria monocytogenes*, LAB, response surface methodology

## Abstract

The objective of this study was to test the effect of the combined application of lactic acid (0–5%) (LA) and UV-C light (0–330 mJ/cm^2^) to reduce *Listeria monocytogenes* and lactic acid bacteria (LAB) on beef without major meat color (L *, a *, b *) change and its impact over time. A two-factor central composite design with five central points and response surface methodology (RSM) were used to optimize LA concentration and UV-C dose using 21 meat pieces (10 g) inoculated with *L. monocytogenes* (LM100A1). The optimal conditions were analyzed over 8 weeks. A quadratic model was obtained that predicted the *L. monocytogenes* log reduction in vacuum-packed beef treated with LA and UV-C. The maximum log reduction for *L. monocytogenes* (1.55 ± 0.41 log CFU/g) and LAB (1.55 ± 1.15 log CFU/g) with minimal impact on meat color was achieved with 2.6% LA and 330 mJ/cm^2^ UV-C. These conditions impaired *L. monocytogenes* growth and delayed LAB growth by 2 weeks in vacuum-packed meat samples throughout 8 weeks at 4 °C. This strategy might contribute to improving the safety and shelf life of vacuum-packed beef with a low impact on meat color.

## 1. Introduction

*Listeria monocytogenes* is a human pathogen that may cause listeriosis, a foodborne infection with a low morbidity and a high mortality rate (20–30%) [1]. The presence of *L. monocytogenes* in raw meat does not cause major public health problems since meat is generally consumed after cooking at temperatures above 70 °C. However, contaminated raw meat when used as raw material for food products that in their production process fail to eliminate the pathogen may present a safety risk [1]. In addition, the presence of *L. monocytogenes* in raw meat constitutes restrictions on international trade.

Contamination of meat with *Listeria monocytogenes* is a consequence of the production process [2]. In addition, *L. monocytogenes* can survive and grow in vacuum-packed meat cuts stored at temperatures between 0 and 4 °C; therefore, different strategies are applied in abattoirs to minimize bacterial contamination [3,4]. Among the different strategies, lactic acid (LA) application is accepted because it does not present risks to consumer health. The maximum concentration of LA allowed is 5% (*m*/*v*) [5]. UV-C light irradiation (UV-C) stands out for its low cost, non-generation of potentially hazardous chemical residues, and low carbon footprint [6]. In addition, UV-C irradiation is an FDA-approved intervention for surface decontamination of foods [7] (FDA, 2019a).

The application of between 2 and 4% LA on meat was reported to reduce *L. monocytogenes* counts on beef surface [8,9]. Reductions between 0.79 to 1.31 log CFU/cm^2^ were obtained in fresh beef when LA was applied from 1% to 4% [9]. Different levels of *L. monocytogenes* reductions have been observed and were associated with factors such as variabilities among strain sensitivity towards stress and forms of LA application [10,11].

UV-C radiation (200 to 280 nm) has been used for decontamination of food surfaces [12]. The ability of UV-C to inactivate *L. monocytogenes* has also been reported, being strain dependent and showing a direct correlation between UV-C dose and *Listeria monocytogenes* reduction [13,14,15]. In addition, UV-C radiation can penetrate the packaging material usually used on meat and meat products such as transparent polypropylene and polyethylene bags [16] and cause significant *L. monocytogenes* reduction on food [13].

Antimicrobial interventions may affect fresh meat color, which is considered to be the single most important characteristic influencing consumer’s purchase decisions [17,18]. Negative effects on meat color are the major problem associated with the use of lactic acid, especially at high concentration [19]. In contrast, UV-C (118–590 mJ/cm^2^) on fresh meat does not appear to cause detrimental color changes [15].

Other bacteria present on the beef surface may be affected by LA and UV-C [15,20]. In refrigerated vacuum-packed meat, lactic acid bacteria (LAB) are the ones that develop the most, being responsible for the production of strong lactic acid off-odors when counts reach 10.000.000 UFC/g at the end of shelf life [21,22,23]. Thus, knowing the effect of LA and UV-C application on LAB may be relevant to improve vacuum packed beef shelf life.

In the past few years, special attention has been given to experimental design and response surface methodology (RSM) to optimize conditions in different systems [24,25]. These modeling tools enable the study of the simultaneous effects of different factors and their interactions on experimental characteristics. This strategy has not been widely used to study the effects of the LA and UV-C combination on vacuum-packed beef. To date, only one report was found describing a similar strategy to study the effects of LA and UV-C on *Salmonella typhimurium* reduction on sliced Brazilian dry-cured loin [26].

In the present work, it was hypothesized that the combined application of LA at low concentrations and UV-C after vacuum packaging might achieve a significant level of reduction in *L. monocytogenes* contamination on beef with a minimal impact on meat color and would contribute to its shelf life by reducing meat LAB counts. To test this hypothesis, a two-factor central composite design and response surface methodology (RSM) were used to optimize the concentration of LA and the UV-C dose applied to vacuum-packed meat that will reduce the amount of *L. monocytogenes* and LAB without significant effects on meat color.

## 2. Materials and Methods

### 2.1. Meat Samples

Eye of round *(Semitendinosus Muscle*) cuts were obtained from a local abattoir. Meat samples were not decontaminated prior to the study. Meat was cut by hand into square pieces of 10 g measuring 5 × 5 cm^2^. Each piece was individually inoculated with *L. monocytogenes* and treated according to the experimental design.

### 2.2. L. monocytogenes Culture Preparation

A strain of *L. monocytogenes* (LM 100A1) previously isolated and characterized in our laboratory was used for this study [27]. The culture was prepared by growing LM 100A1 overnight at 35 °C, to the stationary phase, in tryptic soy broth (Oxoid Ltd., Hampshire, UK) supplemented with 0.6% yeast extract (Oxoid Ltd., Hampshire, UK). The overnight culture was diluted with butterflied buffer to 6.1 log CFU/mL.

### 2.3. Preparation of Lactic Acid Solution

The lactic acid solutions were prepared by diluting a concentrated lactic acid solution (85% *m*/*v*) (PURAC^®^, Corbion, Montevideo, Uruguay) with sterilized distilled water to make 2.5%, 5.0% and 6.0% (*m*/*v*) lactic acid solution. Fresh solutions were prepared prior to each test.

### 2.4. UV-C Irradiation

The specifications of the UV-C lamp used were: 30 W T6 tubular 254 nm with UV germicidal lamp (Code ZW30S19W-Z894, Cnlight Co., Ltd., Guangdong, China), diameter 19 mm, length 894 mm and UV intensity at one meter of 107 µW/cm^2^. Intensity at the application distance was 3.137 mW/cm^2^ measured with a ZED Smart Meter s/N 800,009 (EN61326-1-2013) and the reference sensor D-SICONORM-LP-REF-500 W/m^2^.

Before each trial, the UV-C lamp was preheated for 20 min to stabilize the UV-C emission. UV-C treatments did not increase the surface temperature of the meat to greater than 20 °C.

### 2.5. Experimental Design

A two-factor central composite design with five central points, 2 replicates of factorial points and 2 replicates of axial points were used. The experimental design matrix and all data analysis were performed using Design-Expert^®^ (Version 10, Stat-Ease Inc., Minneapolis, MN, USA). The independent variables were lactic acid concentration (X_1_) and UV-C dose (X_2_), and the dependent variables or response variables were *L. monocytogenes* (LM) log reduction (Y_1_), Lactic Acid Bacteria (LAB) log reduction (Y_2_), and Chroma value (Y_3_). The design matrix consisted of 21 experimental runs including 8 factorial points and 8 axial points with five replicates at the center point (Table 1). Observed responses were fitted to first order, second order and quadratic models. Models were selected by the Sequential Sum of Square Method and assessed based on statistically significant coefficients and *R*^2^ values using ANOVA technique, with a significance level of α = 0.05. For each response variable (Y), a second-order polynomial model equation was defined:

Y = β_0_ + β_1_X_1_ + β_2_X_2_ + β_11_X_1_^2^ + β_22_X_2_^2^ + β_12_X_1_X_2_ (1)

where Y is the measured response of the dependent variables, X_1_ and X_2_ are the independent variables, β_0_ is the intercept, β_1_ and β_2_ are the linear coefficients, β_11_ and β_22_ are the squared coefficients, and β_12_ is the interaction coefficient.

Response surface methodology (RSM) included the generation of 3D response surface and contour plots to study the overall relationships and interactions between independent variables and response factors.

### 2.6. Sample Treatments

According to the experimental design, 21 pieces of 10 g of meat were inoculated with 5.8 log of CFU of the strain LM100A1. Then, 500 µL of the inoculum were disposed on the meat surface and spread with a bent glass rod.

After 10 min, inoculated meat pieces were treated with 1.5 mL of lactic acid solutions from 0 to 6 (*m*/*v*) %. The LA was disposed on the inoculated side, drop by drop covering the entire surface of the meat samples. Then the samples were vacuum packaged in a multi-laminar (EVA, PVDC, PE) thermo-shrinkable bag with a 76% UV-C transmission rate (Cryovac^®^ BB 2620; 50 μm thick, oxygen permeability of 20 cm^3^ m^−2^, 24 h, at 23 °C, and 75% RH; and maximum carbon dioxide permeability of 100 cm^3^ m^−2^, 24 h, at 23 °C, and 75% RH) by use of a vacuum-packaging machine SAMMIC model V-410SGI (Spain).

After packaging, each side of the samples were exposed to 3.137 mW/cm^2^, at 7 cm from the emitting lamp, for 53, 105 and 127 s, achieving doses of UV-C of 165, 330 and 398 mJ/cm^2^ respectively. The UVC-dose range was selected considering the reported UV-C doses applied on beef that did not affect meat color [15], the application conditions, distance from the lamp and duration of the exposures, to be easily implementable in industrial production lines. A second set of samples was prepared for color measurements.

### 2.7. Microbiological Analyses

After treatments, samples were homogenized in sterile bags with 90 mL of Butterfield buffer, appropriate dilutions were plated by duplicate on PALCAM Listeria Selective Agar (Oxoid Ltd., Hampshire, UK) incubated at 37 °C for 48 h for *L. monocytogenes* and on MRS Agar (Oxoid Ltd., Hampshire, UK) incubated anaerobically at 30 °C for 72 h for LAB. Colonies were counted and log transformed. Log reductions of *L. monocytogenes* and LAB per gram of meat compared to samples with no treatments were calculated.

### 2.8. Instrumental Color

At twenty-four hours post treatments, color measurements were performed 30 min after opening the packages. Instrumental lean color (CIE L*: brightness, a*: redness and b*: yellowness) was measured with a Minolta chromameter CR-400 (Konica Minolta Sensing Inc., Tokyo, Japan) using a C illuminant, a 2° standard observer angle and 8 mm aperture size, and calibrated with a white tile before use. Three measurements from each sample were taken and the mean value was calculated. Chroma value was calculated as C * = √ (a*^2^ + b*^2^).

### 2.9. Optimization and Model Validation

The optimized conditions, lactic acid concentration and UV-C dose, were obtained by applying the following constraints on the response factors: (i) to maximize *L. monocytogenes* log reduction; (ii) to maximize LAB log reduction; and (iii) Chroma value > 20, according to MacDougall, et al. 1982 [28] (values above 20 indicate bright red beef).

To validate the proposed model, three experiments were carried out using the optimized conditions as the checkpoint. Experimental responses (log reduction of LM100A1 per gram and log reduction of LAB per gram) of the checkpoint were compared to the predicted results from the fitted models to evaluate the precision of the polynomial equations.

### 2.10. Evolution of L. monocytogenes, LAB, pH and Instrumental Color of LA/UV-C Treated Meat Vacuum Packed and Stored at 4 °C for 8 Weeks

Three experiments were carried out using the optimized conditions (LA 2.6% (*w*/*v*) and UV-C 330 mJ/cm^2^). Meat samples were treated as explained in 2.6., untreated samples were used as a control group. Samples were stored at 4 °C and analyzed for LM, LAB at initial time (week 0) and every two weeks until week 8. Instrumental color was measured at week 0 and week 8. An additional set of samples was prepared for pH determination using a Hanna^®^ model 9025c pH meter with a surface electrode (HI1413B). The T-test was used to compare the data for control and LA/UV-C samples. Data were expressed as mean ± standard deviation. Significance was determined at the *p* < 0.05 level.

## 3. Results

### 3.1. Central Composite Design and Response Surface

*L. monocytogenes* log reduction (Y_1_) varied from no reduction to 1.74 log CFU/g, and LAB log reduction (Y_2_) varied from none to 2.44 log CFU/g. The Chroma value (Y_3_) ranged from 14.51 to 26.90 (Table 1). The analysis of variance (ANOVA) indicates that the best-fitted model (*p* < 0.0001) for *L. monocytogenes* log reduction was the quadratic model, and for LAB log reduction and meat color the best fitted model was the linear (Table 2).

For *L. monocytogenes* log reduction (Y_1_), the quadratic model had a significance of *p* < 0.0001 and an *R*^2^ value of 0.9038, explaining 90.38% of the variability in the response. The similarity between the *R*^2^ and adjusted *R*^2^ values showed the adequacy of the model to predict the corresponding response. The resulting signal-to-noise ratio, measured by the term “adequate precision” (above 4), indicated that the model could be used to navigate the design space (Table 2). The lack of fit of the quadratic model was not significant (F = 0.26, *p* = 0.8522).

For LAB log reduction (Y_2_), the linear model had a significance of *p* < 0.0001, an *R*^2^ value of 0.5774 similar to the adjusted *R*^2^, and a non-significant lack of fit (F = 2.43, *p* = 0.0903). However, the low *R*^2^ value (Table 2) indicates that the model has low precision in the predictions.

The adjusted linear model for meat color (Y_3_) had a significance of *p* < 0.0001 with an *R*^2^ value of 0.8002, similar to the adjusted *R*^2^ (Table 2). The lack of fit of this model was significant (F = 6.74, *p* = 0.0026), suggesting that besides LA and UV-C, there are other factors affecting meat color that were not considered in the experimental design.

For *L. monocytogenes* and LAB log reduction, both LA and UV-C were significant factors. For color, UV-C was not a significant factor in our system. The generated equations for each response, including only the terms with statistical significance (*p* < 0.05), were as follows (Equations (2)–(4)):

Y_1_ = −0.033819 + 0.35890 X_1_ + 6.054 × 10^−3^ X_2_ − 0.043525 X_1_^2^ − 9.17 × 10^−6^ X_2_^2^ (2)

Y_2_ = 0.24423 + 0.24127 X_1_ + 2.04052 × 10^−3^ X_2_ (3)

Y_3_ = 23.63579 − 1.41044 X_1_ (4)

**Figure 1:**
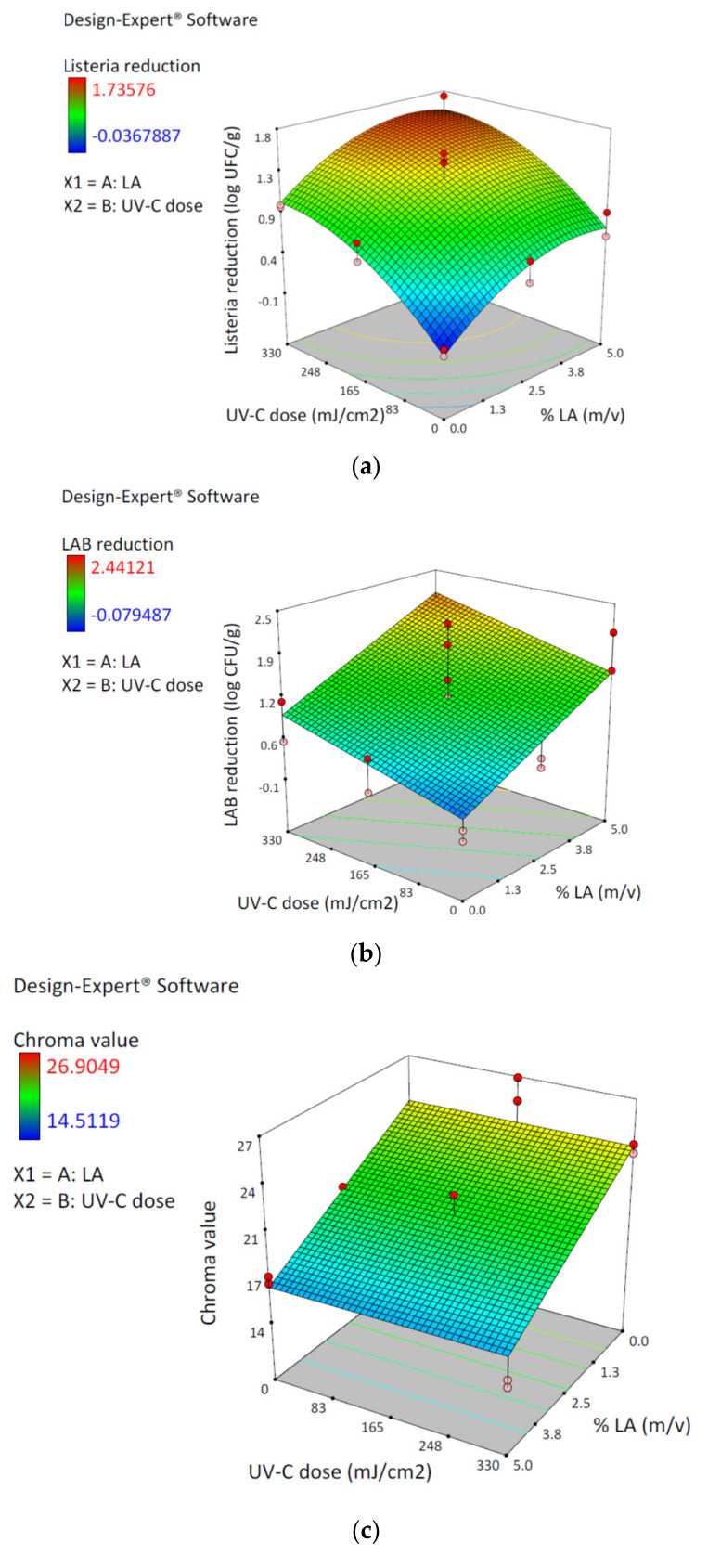
3D response surface plots generated from the Central Composite design showing: (**a**) effect of lactic acid concentration and UV-C dose on *L. monocytogenes* reduction (log CFU/g); (**b**) effect of lactic acid concentration and UV-C dose on LAB reduction (log CFU/g); (**c**) effect of lactic acid concentration and UV-C dose on meat color (expressed as Chroma value).

The 3D response surface plots allow one to visualize the response in the design space (Figure 1). Both LA and UV-C in the ranges studied have a positive effect on LM 100A1 and LAB reduction (Figure 1a,b). For *L. monocytogenes* reduction the 3D response surface plot reflects a curvature according to the quadratic terms in the equation model (Figure 1a). The LAB 3D response surface plot does not present curvature (Figure 1b).

For Chroma value according to Equation (4), the 3D response surface plot showed no changes in Chroma value due to UV-C and a negative effect by LA (Figure 1c).

### 3.2. Optimization and Model Validation

Based on the model generated using the Design Expert software with a desirability factor close to 1, the optimal conditions that satisfy the constraints applied (maximize *L. monocytogenes* reduction; maximize LAB reduction; Chroma value > 20) were: 2.6% lactic acid solution and UV-C dose of 330 mJ/cm^2^. Using these conditions, the model predicted a *L. monocytogenes* reduction of 1.55 ± 0.41 log CFU/g and a LAB reduction of 1.55 ± 1.15 log CFU/g.

Experimental responses using the optimal conditions to treat meat samples were compared to the predicted results from the fitted models to evaluate the precision of the polynomial equations. The experimental values for *L. monocytogenes* and LAB reduction were 1.24 ± 0.18 log CFU/g and 1.20 ± 0.20 log CFU/g respectively. Both *L. monocytogenes* and LAB reduction experimental values were within the 95% CI of the predicted outcome by the models.

### 3.3. Evolution of L. monocytogenes, LAB, pH and Meat Color Treated with 2.6% of LA and 330 mJ/cm^2^ of UV-C Dose Vacuum Packed and Stored at 4 °C for 8 Weeks

#### 3.3.1. *L. monocytogenes* and LAB Counts

**Figure 2:**
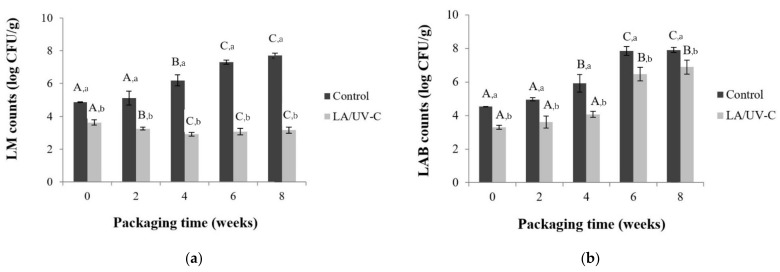
Bacterial count evolution in vacuum packed meats stored at 4 °C (**a**) *L. monocytogenes* (LM) (**b**) Lactic Acid Bacteria (LAB). Light grey and dark grey represent samples treated with 2.6% of LA/330 mJ/cm^2^ of UV-C and no treatment control, respectively. Mean ± SD (*n* = 3) of the values are presented. Different capital letters indicate significant differences at *p* ≤ 0.05 among the means over time for each treatment, and different small letters indicate significant differences at *p* ≤ 0.05 between control and treated samples for each time point.

Application of 2.6% of LA and 330 mJ/cm^2^ of UV-C reduced *L. monocytogenes* and LAB initial log counts by 1.2 and 1.3 log compared to control. Treated meat samples had *L. monocytogenes* and LAB log counts significantly lower (*p* < 0.05) than the control samples throughout the 8 weeks (Figure 2a,b). *L. monocytogenes* counts in LA/UV-C treated meat decreased from 3.6 log to 3.0, while in control samples an increase from 4.86 to 7.38 log CFU/g was observed (Figure 2a). LAB counts in treated samples remained constant until week 4 (*p* > 0.05), then an increase was observed at week 6, reaching 6.89 log CFU/g in week 8. In control samples, LAB counts remained unchanged during the first two weeks, and then increased over time up to 7.85 log CFU/g (Figure 2b).

#### 3.3.2. pH Values

**Figure 3:**
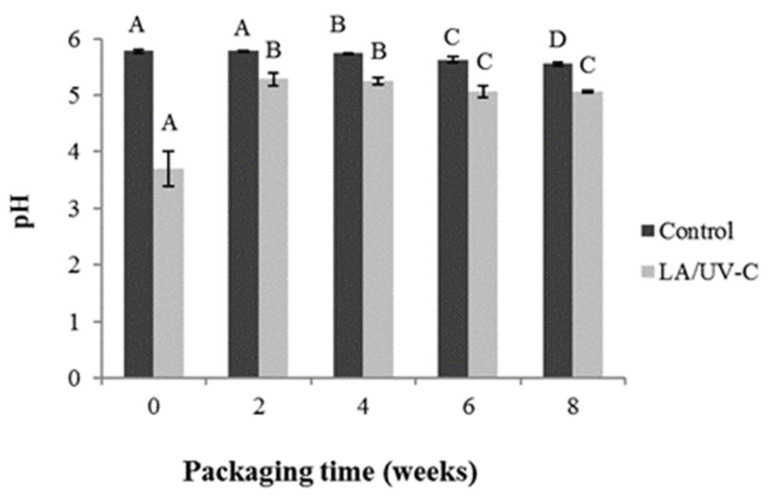
Superficial pH evolution in vacuum packed meats stored at 4 °C. Light grey and dark grey represent samples treated (2.6% of LA and 330 mJ/cm^2^ of UV-C) and control, respectively. Mean ± SD (*n* = 3) of the values are presented. Different letters indicate a significant difference at *p* ≤ 0.05 across time for each treatment.

LA/UV-C treatment decreased (*p* < 0.05) superficial meat pH from 5.78 to 3.70. Then, the treated sample’s pH increased, reaching 5.29 at week 2. After week 2, the superficial pH of treated and untreated samples decreased over time. The pH value of LA/UV-C treated meat was always lower (*p* < 0.05) than the control. The final pH for control samples was 5.55 and for treated samples was 5.07 (Figure 3).

#### 3.3.3. Color Measurements

Changes in CIE L*, a*, b* and Chroma values (C*) at weeks 0 and 8 are shown in Table 3. At the initial time, L*, b* and C* values of LA/UV-C treated and untreated meat did not show variations from each other (*p* > 0.05); the a* value of control meat was higher (*p* < 0.05) than the value of LA/UV-C treated meat. At week 8, treated and untreated meat had non-significant differences in b* and C* values. However, the L* and a* values of LA/UV-C treated meat were lower (*p* < 0.05) than the control.

For both control and LA/UV-C treated meat, CIE L*, a*, b* and C* values were lower (*p* < 0.05) at 8 weeks compared to values at week 0, except the L* value of control samples that showed no significant change (*p* > 0.05).

## 4. Discussion

The present study shows for the first time the effect of the combined application of LA and UV-C on *L. monocytogenes* and LAB in vacuum-packed beef. A central composite design and Response Surface Methodology were used to optimize the concentration of LA and the dose of UV-C to reduce the population of *L. monocytogenes* and LAB without major changes in meat color.

The major findings of the present study were: (i) the quadratic model obtained allowed us to predict *L. monocytogenes* log reduction in vacuum-packed beef treated with LA and UVC, (ii) the maximum log reduction for both *L. monocytogenes* (1.55 ± 0.41 log CFU/g) and LAB (1.55 ± 1.15 log CFU/g) with minimal impact on meat color was achieved with the application of 2.6% LA and 330 mJ/cm^2^ UV-C, and (iii) under these conditions, there was no increase in *L. monocytogenes* counts over 8 weeks of storage at 4 °C, and LAB growth was delayed by 2 weeks compared to control samples.

In the present study, the quadratic model obtained for predicting inoculated *L. monocytogenes* reduction had a good predictor value (*R*^2^ = 0.9038). Both LA (0–5%) and UVC (0–330 mJ/cm^2^) were significant (*p* < 0.05) factors and had independent effects (no significative interaction (*p* > 0.05)). The non-significant interaction between the factors indicated that the effects were additive, observed also for *Salmonella* inactivation in a different food matrix [26]. The reduction in *L. monocytogenes* increased as LA concentration and UV-C dose increased. According to the model, the combination of the maximum levels of LA (5.0%) and UV-C (330 mJ/cm^2^) reduced the 5.8 log inoculum by 1.73 log, a fraction of viable microorganisms remained in the sample, indicating the presence of a tailing effect. This is depicted in the 3D Response Surface plot (Figure 1a), where *L. monocytogenes* log reduction had an initial sharp increasing rate and then decreased at higher UV-C doses and LA concentrations. As mentioned before, there are no other studies reporting the combined action of LA and UV-C on *Listeria monocytogenes* in fresh meat, although similar inactivation patterns were observed in fresh beef treated separately with LA or with UV-C [9,15,27]. In this respect, a previous study from our group obtained a reduction of 1.13 log using 2.5% LA. DeGeer et al. 2016, using a 4% LA solution, reduced by 1.3 log a *L. monocytogenes* inoculum of 8 log and, Kalchayanand et al. 2020, using a 590 mJ/cm^2^ UV-C dose, reduced a 6 log *L. monocytogenes* inoculum by 0.89 log. The observed tailing effect for *L. monocytogenes* inactivation in meat may be explained by the ability of the meat matrix to buffer the antimicrobial solution and to entrap *L. monocytogenes* into muscle fibers shielding the bacteria from LA and UV-C radiation [9,29].

LAB reduction by LA and UV-C was adjusted to a linear model in which the factors LA and UV-C were both significant (*p* < 0.05) and independent (no significative interaction (*p* ˃ 0.05)). The low precision (*R*^2^ = 0.5774) of the model for predicting the response in the design space was a consequence of the variability among the five replicas of the central point (Table 1). This variability may be attributed to the natural diversity of the LAB present in the meat samples, which may have different sensitivity to LA and UV-C [14,20]. The maximum level of LAB reduction matched with the highest LA concentration and UV-C dose used suggesting that both factors can be further increased to achieve a higher level of reduction as shown in the 3D Response Surface plot (Figure 1b).

However, it was not feasible to increase LA concentration because (i) regulations of USDA/FSIS and European Commission do not allow concentrations greater than 5%, and (ii) high LA concentrations produced unwanted color changes in meat. Regarding increasing the UV-C dose, our data suggested that an increase in UV-C dose would not achieve a larger reduction in the *Listeria monocytogenes* population. Though our model did not allow us to predict outside the design space, the level of *L. monocytogenes* reduction obtained at the +α experimental point applying 2.5% LA and 368 mJ/cm^2^ (Table 1) was similar to the level of reduction achieved with 2.5% LA and 330 mJ/cm^2^. In agreement with this observation, McLeod, et al., 2017 [29] reported that the application of 3 J/cm^2^ did not increase the level of *Listeria monocytogenes* reduction in chicken breast beyond the reduction level obtained with 0.3 J/cm^2^. The antilisterial effect of doses higher than 330 mJ/cm^2^ combined with 2.5% LA needs to be further studied, as well as the effects on meat quality.

Meat color change, expressed as Chroma value, was only related to LA acid concentration, and was fitted to a linear model with one factor (Figure 1c). Chroma value detrimental change was mostly due to the decrease in redness value (a*) (Table S1, Supplementary File), a well-known effect of lactic acid in beef [19]. The fact that UV-C doses applied did not have a significant effect on fresh meat color was in agreement with previous studies [15].

Using the models obtained for each response (*L. monocytogenes* reduction, LAB reduction and Color), RSM predicted that 2.6% LA concentration and 330 mJ/cm^2^ of UV-C dose were the conditions that combined satisfied the constraints imposed (highest *L. monocytogenes* and LAB reduction and chroma value equal or larger than 20). The *L. monocytogenes* reduction predicted was 1.55 log. This reduction level was higher than the reduction levels obtained with the highest LA concentration or the highest UV-C dose alone.

Treatment with 2.6% LA and UV-C of 330 mJ/cm^2^ at the time of packaging prevented the surviving fraction of the inoculated population of *L. monocytogenes* from thriving, showing a tendency to decrease with time when stored at 4 °C. In the untreated samples, the counts of *L. monocytogenes* increased (Figure 2a). LA and UV-C caused cellular injury in the fraction of survivors preventing them from overcoming the additional stress imposed by low oxygen and temperature. There are no reports of the combined application though a similar trend was reported for *L. monocytogenes* over time in beef treated with LA [27,30]. Regarding the behavior of *L. monocytogenes* in control samples, previous studies reported both growth [31,32] and inhibition [27,30,33] during storage at 4 °C. Differences in *L. monocytogenes* behavior may be due to variations in experimental conditions such as moisture and pH of meat samples, oxygen permeability of the vacuum bags and the *L. monocytogenes* strains used. In this study, the strain used was isolated from a refrigerated environment after having suffered different types of stress which, according to Skandamis et al., 2008, may affect subsequent stress tolerance.

LAB followed a sigmoidal growth curve [22]. Treatment prolonged the lag phase by two weeks, probably because a fraction of the remaining living cells were injured by UV-C and would have a slower growth rate or would be unable to replicate under stress [14]. The final LAB count in control samples reached 8 Log while in the samples treated with LA/UVC reached 7 Log. Though these results were relevant, more studies are needed to understand the impact on the combined LA/UV-C application on meat shelf life.

At time zero, redness was the only color component that was affected by the combined LA/UV-C application. As mentioned above, the decrease in initial a* is mainly due to the application of LA [19,34]. After eight weeks of storage, treated samples had different values of L* and a* with respect to samples without treatment, however the chroma value was similar in both samples. The greater decrease in the value of L* and a* at week 8 in the treated samples may be due to the fact that both LA and UVC have the capacity to oxidize myoglobin and to cause lipid oxidation with the consequent loss of color in meat [26,35]. However, more studies need to be done to assess the color of treated meats over time.

In summary, the combined application of LA 2.6% and UV-C 330 mJ/cm^2^ contributed to improving safety of vacuum packed beef, with a low impact on color. Although, more studies must be carried out regarding the effects on other bacteria present on meat and other physicochemical changes such as lipid and protein oxidation.

## 5. Conclusions

The selected Central Composite design and response surface methodology were effective tools to optimize and study the effects of LA and UV-C parameters on the *L. monocytogenes* and LAB reduction in vacuum packed beef. The combined application of LA and UV-C radiation under the tested conditions proved to be a useful strategy to reduce L. *monocytogenes* and LAB population in meat without significantly affecting meat color. The treatment had an effect over time by preventing *L. monocytogenes* growth and delaying LAB growth. The latter might have an impact on vacuum beef shelf life. The maximum reduction on *L. monocytogenes* obtained without significant changes in color was 1.55 log CFU/g. Considering that the usual amount of *L. monocytogenes* in fresh meat is low, this level of reduction is significant for meat safety purposes.

## Figures and Tables

**Table 1:** Central composite experimental design matrix and observed responses.

Runs	% Lactic Acid (*m/v*) (X_1_)	UV-C Dose (mJ/cm^2^) (X_2_)	Reduction LM (Log CFU/g) (Y_1_)	Reduction LAB (Log CFU/g) (Y_2_)	Chroma Value (Y_3_) ^a^
1	2.5	398	1.59	2.44	21.48
2	0.0	165	0.62	0.66	26.90
3	2.5	0	0.38	0.45	19.14
4	2.5	0	0.63	0.59	20.25
5	2.5	165	1.20	1.13	18.69
6	5.0	330	1.74	1.14	15.02
7	5.0	330	1.34	1.70	14.51
8	5.0	0	0.58	1.48	17.34
9	2.5	165	1.53	1.11	18.73
10	0.0	0	0.04	0.08	22.57
11	2.5	165	1.43	2.25	21.61
12	2.5	398	1.45	1.67	20.61
13	0.0	165	0.82	0.15	25.24
14	6.0	165	1.49	2.24	16.60
15	0.0	0	−0.04	−0.08	22.77
16	5.0	0	0.85	2.06	16.82
17	2.5	165	1.08	1.39	18.69
18	6.0	165	0.96	1.63	17.43
19	0.0	398	0.92	0.49	23.82
20	0.0	398	0.94	1.12	23.20
21	2.5	165	1.42	1.94	18.73

^a^ Mean of three values per sample.

**Table 2:** Summary of the regression analysis of the three responses.

Response	Model	Significance	*R* ^2^	Adjusted *R*^2^	Predicted *R*^2^	Adequate Precision
Y_1_	Quadratic	<0.0001	0.9038	0.8718	0.8111	16.646
Y_2_	Linear	<0.0001	0.5774	0.5304	0.4329	9.563
Y_3_	Linear	<0.0001	0.8002	0.7780	0.7314	14.571

**Table 3:** Instrumental color parameters (L*, a*, b*) measured and Chroma value (C*) treated LA/UV-C and control meat samples at initial time and at 8 weeks.

Time (Weeks)		Control	LA/UV-C
0	Lightness (L*)	47.69 ± 2.94 ^a^	46.71 ± 3.51 ^a^
	Redness (a*)	20.21 ± 2.85 ^a^	15.43 ± 1.92 ^b^
	Yellowness (b*)	13.07 ± 0.77 ^a^	13.36 ± 0.63 ^a^
	Chroma (C*)	24.10 ± 2.48 ^a^	20.44 ± 1.56 ^a^
8	Lightness (L*)	46.94 ± 2.71 ^a^	40.95 ± 1.81 ^b^
	Redness (a*)	13.53 ± 1.63 ^a^	9.92 ± 0.69 ^b^
	Yellowness (b*)	11.08 ± 0.27 ^a^	11.62 ± 0.56 ^a^
	Chroma (C*)	17.52 ± 1.14 ^a^	15.28 ± 0.66 ^a^

Different superscripts within a row show significant results (*p* ≤ 0.05). Data recorded as Mean ± Standard Deviation.

## Data Availability

Not applicable.

## Supplementary Materials

The following are available online at https://www.mdpi.com/article/10.3390/foods10061217/s1, Table S1: Central composite experimental design matrix with L*, a* and b* values for beef samples.
