# Supplementary material for: Effect of UV-C Irradiation and Lactic Acid Application on the Inactivation of Listeria monocytogenes and Lactic Acid Bacteria in Vacuum-Packaged Beef
Source: Foods. 2021 May 28;10(6):1217. doi: 10.3390/foods10061217 (PMC8226716; doi:10.3390/foods10061217)
Supplement: Supplementary file 1 [file foods-10-01217-s001.zip › foods-1225860-supplementary.pdf]

Table S1. Central composite experimental design matrix with the L*, a* and b* values recorded for beef samples.

Runs	% Lactic acid (m/v) (X_1)	UV-C dose (mJ/cm²) (X_2)	Lightness (L*)[a]	Redness (a*)[a]	Yellowness (b*)[a]
1	2.5	398	48.88	16.74	13.46
2	0.0	165	45.57	23.12	13.76
3	2.5	0	54.8	14.42	12.59
4	2.5	0	52.57	15.35	13.21
5	2.5	165	53.14	13.18	13.25
6	5.0	330	43.22	10.13	11.09
7	5.0	330	44.14	9.89	10.62
8	5.0	0	45.41	11.74	12.76
9	2.5	165	54.73	12.49	13.96
10	0.0	0	50.26	17.97	13.65
11	2.5	165	53.94	12.85	13.61
12	2.5	398	43.67	15.95	13.05
13	0.0	165	49.03	21.23	13.65
14	6.0	165	48.33	10.09	13.18
15	0.0	0	48.33	19.23	12.19
16	5.0	0	50.51	10.59	13.07
17	2.5	165	53.50	12.92	13.51
18	6.0	165	41.87	12.25	12.4
19	0.0	330	48.74	20.64	11.89
20	0.0	330	47.83	19.66	12.31
21	2.5	165	54.02	13.03	13.45

[a] Mean of three values per sample.
